# In which developing countries are patents on essential medicines being filed?

**DOI:** 10.1186/s12992-017-0262-4

**Published:** 2017-06-26

**Authors:** Reed F Beall, Rosanne Blanchet, Amir Attaran

**Affiliations:** 1 Population Health Program, University of Ottawa, One Stewart St, Rm 221, Ottawa, ON K1N 6N5 Canada; 2 Faculties of Medicine, University of Ottawa, One Stewart St, Rm 221, Ottawa, ON K1N 6N5 Canada; 3 Faculties of Law, University of Ottawa, One Stewart St, Rm 221, Ottawa, ON K1N 6N5 Canada; 4 Interdisciplinary School of Health Sciences, University of Ottawa, 35 University Private, THN 050, Ottawa, ON K1N 6N5 Canada; 5 Present Address:, 1620 Tremont St., Suite 3030, Boston, MA 02120 USA

**Keywords:** Pharmaceutical policy, Intellectual property, Access to medicines, Essential medicines, Global governance, International trade

## Abstract

**Background:**

This article is based upon data gathered during a study conducted in partnership with the World Intellectual Property Organization on the patent status of products appearing on the World Health Organization’s 2013 Model List of Essential Medicines (MLEM). It is a statistical analysis aimed at answering: in which developing countries are patents on essential medicines being filed?

**Methods:**

Patent data were collected by linking those listed in the United States and Canada’s medicine patent registers to corresponding patents in developing countries using two international patent databases (INPADOC and Derwent) via a commerical-grade patent search platform﻿ (Thomson Innovation). The respective supplier companies were then contacted to correct and verify our data. We next tallied the number of MLEM patents per developing country. Spearman correlations were done to assess bivariate relationships between variables, and a multivariate regression model was developed to explain the number of MLEM patents in each country using SPSS 23.0.

**Results:**

A subset of 20 of the 375 (5%) products on the 2013 MLEM fit our inclusion criteria. The patent estate reports (i.e., the global list of patents for a given drug) varied greatly in their number with a median of 48 patents (interquartile﻿ range [IQR]: 26-76﻿). Their geographic reach had a median of 15% of the developing countries sampled (IQR: 8-28%). The number of developing countries covered appeared to increase with the age of the patent estate (*r* = .433, *p* = 0.028). The number of MLEM patents per country was significantly positively associated with human development index (HDI), gross domestic income (GDI) per capita, total healthcare expenditure per capita, population size, the Rule of Law Index, and average education level. Population size, GDI per capita, and healthcare expenditure (in % of national expenditure) were predictors of the number of MLEM patents in countries (*p* = 0.001, *p* = 0.001, *p* = 0.009, respectively). Population size was the most important predictor (β = 0.59), followed by income (GDI per capita) (β = 0.32), and healthcare expenditure (β = 0.15). Holding the other factors constant, (i) 14.3 million more people, (ii) $833.33 more per capita (GDI), *or* (iii) 0.88% more of national spending on healthcare resulted in 1 additional essential medicine patent.

**Conclusion:**

Population was a powerful predictor of the number of patent filings in developing countries along with GDI and healthcare expenditure. The age and historical context of the patent estate may make a difference in the number of patents and countries covered. Broad surveillance and benchmarking of the global medicine patent landscape is valuable for detecting significant shifts that may occur over time. With improved international medicine patent transparency by companies and data available through third parties, such studies will be increasingly feasible.

**Electronic supplementary material:**

The online version of this article (doi:10.1186/s12992-017-0262-4) contains supplementary material, which is available to authorized users.

## Background

The World Health Organization’s (WHO) Model List of Essential Medicines (MLEM) [1] identifies effective health products that all persons should, at a bare minimum, have access to, regardless of where they live or how much money they have. Updated biennially, the products appearing on the MLEM are typically older and off-patent. However, over the past 15 years, several newer medicines for treating HIV and other diseases have been added to the list [2]. As these medicines were both patented and expensive, friction arose between advocates of intellectual property protection and advocates of essential medicine access [3]. These debates continue today and extend beyond HIV to other disease burdens, such as hepatitis C and cancer [4, 5].

Price is an important determinant of access to essential medicines [6]. As prices may be elevated where patents are protected, there has been concern that patent protection may indirectly impact access [7]. Others have argued that more market launches occur sooner where patents exists, thereby facilitating access [8, 9]. Either way, patents have potential to indirectly influence medicine access. It is therefore important to monitor the fundamental matter of where manufacturers have filed patents on MLEM products globally [10, 11]. There have been many medicine patents landscape studies with relatively narrow focus upon one or more specific medicines [12–18], upon a limited sample of purposefully-chosen developing countries, and upon specific types of patents on aspects of the medicines that are more likely to block generic competition (e.g., compound patents). Fewer have taken broader scope, surveying relatively larger sets of medicines and developing countries to provide an overview of the landscape [19, 20].

While both approaches hold value, the current undertaking assumes a wider scope. It is an update in a series of studies that survey the essential medicine patent landscape in developing countries using contemporaneous editions of the MLEM (i.e., 2004 [21], 2009 [22], and 2011 [23]), on a large portion of the world’s developing countries, and on patents pertaining to any aspect of the medicine (e.g., formulation, method of use). This article details statistical analyses performed upon the data collected during the fourth study to follow in this tradition of surveying the entire essential medicine patent landscape, which used the 2013 MLEM and was published by the World Intellectual Property Organization (WIPO) in April 2016 [24, 25]. This study built upon its predecessors by increasing the developing country sample size (2004: 65; 2009: 9; 2011: 95; current study: 137) and by verifying the data with supplier companies (in contrast to the 2009 and 2011 studies), making it the largest study on the essential medicine patent landscape to our knowledge. The present article’s research question is: in which developing countries are patents on essential medicines being filed? This study also aims at describing the size and geographic reach of MLEM patent estates in developing countries over time.

## Methods

The international patent data collection was completed in three phases: (i) identifying which medicines from the MLEM could be considered “patented” using the United States Food and Drug Administration’s Orange Book [26], Health Canada’s Patent Register [27] and Drug Product Database [28], and previous studies [21–23]; (ii) linking these patent data to related patents abroad using international patent databases (INPADOC [29] and Derwent [30] via Thomson Innovation [31]) and creating a preliminary landscape report; and finally (iii) approaching each medicine supplier with our preliminary data and requesting their feedback. We did not discriminate or distinguish between patent types (e.g., compound, formulation, method of use, process); all patents that were identified through the data collection and verification process were included. However, our focus was upon developing countries, and therefore, we excluded those categorized by the World Bank as “High” income or as having “Very High” development designation by the United Nations [32, 33]. Nine of 11 companies participated in the study. The fieldwork was undertaken in September 2014 – May 2015 using the 2013 MLEM as it was the latest available when the study commenced. More details on our methodology for this phase of the study are available in the web supplement (see Additional file 1) and in the WIPO report [25].

With the data returned from supplier companies, we conducted a concordance study to understand how their records compared to what we had found in international patent databases. This full analysis is published elsewhere [34, 35]. Some disagreement between these lists is expected because international patent databases are not updated in real time and their reach into many developing countries is limited. This is why verification procedures are important in landscape studies like this one. The agreement of the product-patent-country combinations between the two datasets was overall reasonably good at 83% (1060/1280), though its precision varied widely by product. As for the disagreement between the datasets (*n* = 220), 57% (*n* = 126) were instances wherein our report showed a patent record, but the companies’ records did not. Conversely, 43% (*n* = 94) were instances wherein companies’ records showed a patent filing, but ours did not. In instances of discrepancies, data from supplier companies were taken for the current study because it was deemed more up-to-date.

We organized the combined data into a table with the MLEM medicines as the row headers (horizontally) and the developing countries as the column headers (vertically), and then tallied number of patents relevant to each drug-country combination. This data table served as the basis for our statistical analyses. This table is made available as an excel file in the web supplement (see Additional file 2) and in the WIPO report [25].

Statistical analyses were carried out using SPSS 23.0 [36]. A *p* value <0.05 was considered significant. A one-tailed Spearman correlation analysis was performed to assess whether the age of the medicine’s patent estate (i.e., the global list of patents for a given drug) was associated with the number of countries listed in them. To do so, we retrieved the first marketing approval date for the medicines in our sample from the Orange Book [26] (or the Purple Book for biologics [37]) as well as the first publication date listed in the Merck Index [38] (a scientific reference publication that lists patents of therapeutic compounds). Spearman correlations (two-tailed) were also done to assess bivariate relationships between the number MLEM patents per country, the number of MLEM drugs patented per country, and the development indicators. A multivariate regression model was developed to explain the number of MLEM patents in each country. Robust multivariate regressions were performed using the bootstrap procedure to ensure accurate and generalizable results because some variables were not normally distributed [39]. Variables to be included in the model were selected a priori on a theoretical basis to avoid over-fitting the data [39]. Indeed, the model was built upon previous evidence suggesting that patent filings follow high-value national markets [21, 22]. We focused on commonly used development indicators which were widely available for the countries contained in the sample [21–23]. Selected variables were population size (in millions), gross domestic income (GDI) per capita, and total healthcare expenditures (as a percentage of gross domestic product [GDP]) [33, 40]. Total healthcare expenditures as a percentage of GDP was chosen instead of total healthcare expenditures per capita because of multicollinearity between this last indicator and GDI per capita. Absence of multicollinearity between independent variables was assessed using a variance inflation factor test. In additional models, we further adjusted for the Gini Index [41], and the Rule of Law Index [42], separately. The Rule of Law Index is a composite score compiled by the World Justice Project and may be suggestive of the general strength of countries’ legal systems, though intellectual property law is not given specific consideration [42]. As patents confer the right to patent holders to pursue financial compensation from infringers in law courts, we hypothesized companies may be more likely to file patents in countries where the legal system is strong (and the Rule of Law Index score is accordingly high). In both cases, the additional determinant (either Gini Index or the Rule of Law Index) was not significant, other determinants were similarly associated with the outcome and we lost some statistical power (n decreased from 129 to 60 and 69, respectively). Therefore, we present only the original model here. Standardized beta values were used to provide insight into the relative importance of predictors in the model [39]. These values tell us the number of standard deviations that the outcome (number of MLEM patents) will change as a result of one standard deviation change in a predictor (population, GDI per capita *or* healthcare expenditures) [39].

### Disclaimer

As stated above and elsewhere [25], most of the patent data discussed in this study have been verified by the companies. However, because of the temporally dynamic nature of the legal status of patents (e.g., patent applications can be granted belatedly or granted patents can be invalidated), a small number of errors are likely to remain even after several rounds of verification, although not so many as to materially affect our conclusions. While satisfactory for an academic study, we stress that these data are not sufficient for evaluating legal freedom to operate for commercial purposes. Given the serious legal consequences of patent infringement, no promise or warranty as to the accuracy of the data is made or implied, and we strongly recommend that anyone wishing to rely on these findings obtain independent legal advice before doing so.

## Results

Our sample included 20 of the 375 drugs (or about 5%) on the 2013 MLEM which had some kind of patent protection in at least one developing country. Across the 20 drugs and 137 countries, we found 1735 patent filings overall. Most of these medicines were for HIV (13 of 20); the remaining ones were antibiotics, other anti-virals, or were for non-communicable diseases (cancer and gastroesophageal reflux). The web supplement lists our sample of medicines and where patents were located (see Additional file 2). One-third of the countries (*n* = 44) had no essential medicine patent filings whatsoever. Oppositely, Table 1 shows the top-10 countries where we found the most essential medicine patent filings.

**Table 1:** Top-10 developing countries for MLEM patent filings

1. China 2. Mexico 3. Romania 4. Philippines 5. Bulgaria	6. Brazil 7. Turkey 8. India 9. South Africa 10. Indonesia

### Size and geographic reach of the patent estates over time

We found that the 20 patent estate reports varied greatly in their number with a median of 48 patents, a range of 1–173 patents, and an interquartile range (IQR) of 26–76 patents. Their geographic reach had a median of 15%, a range of <1–44%, and an IQR of 8–28% of developing countries. The marketing approval dates according to Orange Book [26] (or Purple Book [37] for biologics) ranged from 1987 to 2016 (median: 2003; IQR: 1998–2007). We found a statistically significant positive correlation between the number of countries listed in the patent estates and their age based on the first marketing approval date recorded in the Orange Book (or Purple Book) (*r* = 0.433; *p* = 0.028). On the other hand, when we used the patent publication date listed in the Merck Index [38] to date the patent estates, we found no correlation.

### Where are MLEM patents being filed?

Correlations between the number MLEM patents per country, the number of MLEM drugs patented per country, and development indicators are presented in Table 2. As expected the number of MLEM patents and the number of MLEM drugs patented per country were highly associated (*r* = .995; *p* < 0.0001). Consequently, and for brevity, we only discuss the number of MLEM patents per country from now on. The number of MLEM patents per country was significantly positively associated with population size, GDI per capita, total healthcare expenditures per capita, HDI, average education, and the Rule of Law Index, but it was not associated with the Gini Index, total healthcare expenditures (as a percentage of GDP), or life expectancy at birth.

**Table 2:** Spearman correlation coefficients (*r*) between MLEM patents and development indicators

	Total MLEM patents	MLEM drugs patented	HDI	Life expectancy at birth	Average education	Expected education	GDI per capita	Total healthcare expenditure per capita	Total healthcare expenditure % of GDP	Population	Gini	Rule of lax index
Total MLEM patents	1	.995***	.232**	.103	.268**	.163 ns	.241**	.256**	.121	.441***	.102	.326**
MLEM drugs patented	137	1	.255**	.133	.277**	.179*	.267**	.276**	.114	.457***	.129	.327**
HDI	129	129	1	.831***	.839***	.855***	.917***	.873***	−.026	−.142	−.087	.480***
Life expectancy at birth	129	129	129	1	.579***	.655***	.711***	.716***	.020	−.130	.011	.325**
Average education	129	129	129	129	1	.694***	.688***	.730***	.035	−.217*	−.032	.467***
Expected education	129	129	129	129	129	1	.730***	.708***	.010	−.144	.010	.454***
GDI per capita	129	129	129	129	129	129	1	0.875***	−.094	−.067	−0.60	.462***
Total healthcare expenditure per capita	127	127	125	125	125	125	125	1	.263**	−.225*	.121	.447***
Total healthcare expenditure % of GDP	128	128	125	125	125	125	125	127	1	−.178*	.295*	.050
Population	134	134	129	129	129	129	129	127	128	1	−0.36	−.320**
Gini	60	60	60	60	60	60	60	60	60	60	1	.018
Rule of lax index	69	69	69	69	69	69	69	67	67	69	43	1

*p < 0.05, **p < 0.01, ***p < 0.001

Table 3 presents the multivariate regression model predicting the number of MLEM patents per country. Population size was the most highly significant positive predictor of the number of MLEM patents per country (*p* = 0.001), followed by wealth (GDI per capita; *p* = 0.001), and total healthcare expenditure (as a proportion of the GDP; *p* = 0.009).

**Table 3:** Linear regression model of predictors of number of MLEM patents in developing and emerging countries (*n* = 129)

	*b* ^a, b^	SE Β^2^	β	*p*	R^2^
Constant	−5.95 (−14.10, 1.31)	4.45			
Population (in million)	0.07 (0.05, 0.20)	0.01	.59	*p* = 0.001	
GDI per capita (in 1000$)	1.20 (0.55, 1.77)	0.26	.32	*p* = 0.001	
Total healthcare expenditure (% of GDP)	1.14 (0.38, 2.16)	0.54	.15	*p* = 0.037	
Model R^2^					0.44

^a^95% bias corrected and accelerated confidence intervals reported in parentheses

^b^Confidence intervals and standard errors based on 1000 bootstrap samples

Based on standardized beta values, population size was the most important predictor of the number of patents in a country (β = 0.59). Holding the other two factors constant (income and healthcare expenditure), an increase of 1 million persons was associated with an increase of 0.07 essential medicine patent filings. GDI per capita was the second strongest predictor and was about half as strong as the population variable (β = 0.32). An increase of $1000 GDI per capita was associated with an increase of 1.2 essential medicine patent filings when holding the other two factors constant (population and healthcare expenditure). The healthcare expenditure variable was the least strong predictor and was about half as strong as was GDI per capita (β = 0.15). Holding the other two factors constant (population and income), an increase of 1% of GDP spent on healthcare was associated with an increase of 1.14 essential medicine patent filings. In other words, holding the other factors constant, (i) 14.3 million more people, (ii) $833.33 more income per capita (GDI), *or* (iii) 0.88% more of national spending on healthcare resulted in 1 additional essential medicine patent. This model accounts for 44% of the variance in the number of MLEM patents per country (r^2^ = 0.44). The regression equation is:

$$ \mathrm{Number}\ \mathrm{of}\ \mathrm{MLEM}\ \mathrm{patents}=-5.95+\left(0.07\mathrm{x}\ \mathrm{population}\right)+\left(1.20\mathrm{y}\ \mathrm{GDI}\right)+1.14\mathrm{z} $$

Where: (total health care expenditure)

- x = population size (in million)
- y = GDI per capita (in $1000)
- z = total healthcare expenditure (in percent of GDP)

## Discussion

This study has investigated what factors might influence where companies direct their patent filings on essential medicines in the developing world. We found that (i) older patent estates in our sample tended to cover more countries than newer ones, and that (ii) countries with larger potential market size (large populations with higher wealth and healthcare spending) tended to attract more patent filings. We discuss these results in more detail below, including how these compared to previous study completed a decade ago [21]. We compare with the 2004 study [21] because its results are available in the published literature (the 2009 and 2011 studies are not). Further, we followed a similar verification procedure to the 2004 study, which increases the comparability of the findings.

### Size and geographic reach of the patent estates over time

The 2004 study found at least one filing in a median of 18% of the developing countries sampled (17 medicines/65 countries; IQR: 11–32%). This was somewhat larger than present findings (median 15%; 20 medicines/137 countries; IQR: 8–28%), especially those patent estates surveyed with the relatively highest coverages. The maximum coverage in the current study was 44% of developing countries (pegylated interferon alfa 2a), whereas four medicines were above this threshold in 2004 (abacavir: 74%; lamivudine: 71%, nevirapine: 61%, and nelfinavir: 48%).

**Fig. 1:**
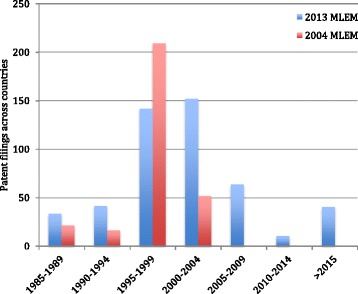
Essential medicine patent filings across medicine sample in developing countries

These size differences may be explained by the patent estates’ ages at the time of these studies. Those in 2004 were notably newer at the time (median time since US market entry: 8 years; IQR: 6–10 years) than in the current study (median: 12 years; IQR: 7–17 years). While we report above the positive correlation (*r* = 0.433; *p* = 0.028) between patent estates’ ages based on the Orange Book (or Purple Book) and their number of patents, we did not find the same relationship when re-analyzing the data from the 2004 study (in fact, we found a tendency in the opposite direction [*p* = 0.085]). Similarly, we did not find an association in the data from the 2004 study when the age of patent estates was based on Merck Index [38]. This discrepancy may be indicative that marketing approval dates are causing an artifact, or that they are a better indicator for when secondary patenting activity may become accelerated [43]. Further investigation of both data showed that medicines which came to market between 1995 and 2005 had an especially high number of patent filings. This is illustrated in Fig. 1. This apparent high period for medicine patent filing activity may explain why the direction of the correlation seemed to have shifted between the two studies.

There are several reasons rooted in the history and politics of 1995–2005 that could explain why there may have been uniquely high patent filing activity in developing countries at that time. The Trade-related Aspects of Intellectual Property Agreement (TRIPS) was instituted in 1995 [44], which initiated a period of patent law synchronization amongst its 147 member states. Also, this same timeframe was a significant breakthrough period for HIV treatments (which constitutes many of the medicines in our sample). Further, this was when the global HIV epidemic began to draw serious attention from high-income countries and when many medicine access campaigns were launched. These dynamics and unprecedented pressures may have led many drug makers to become especially active in filing patents in developing countries. Still, several of the patent estates (didanosine, efavirenz, lopinavir + ritonavir, ritonavir) stemming from this high period appeared to be larger in the current study than in 2004, which may simply be due to patent filing on secondary aspects of the medicines (e.g., heat-stable formulations), or unusually large lags in patent filing times between developing countries that were not yet reflected in the 2004 study’s data.

### Where are MLEM patents being filed?

While our study corroborates previous findings regarding the importance of economic indicators in predicting where higher numbers of patents are filed in the developing world [3, 21–23], population size was the most powerful predictor in our model. This brings an additional layer of nuance to the previous patent surveys on the MLEM, as they focused more predominately on economic indicators. Studies from other sectors and contexts have also observed concentrations of patents corresponding to concentrations of people [45]. Indeed, we found that a disproportionally high share of the patents were in the developing world’s most populated countries, though there are some major exceptions, such as Bangladesh. For instance, India, Indonesia, and the Philippines appear in the top-10 list in Table 1, even though they have relatively low incomes per capita.

This finding regarding population size is an occasion to underscore that the scale of patent estates’ geographic coverage is not necessarily indicative of their potential to influence medicine access from a global population health perspective because populations are not distributed equally across countries. Others have made this objection previously in the literature [46, 47]—a point that may eventually become more urgent now that countries like India have adjusted their patent laws to be TRIPS-compliant (even though there has only been one compulsory license for export from Canada to Rwanda). For instance, two effective patents in India and China alone pertains to a population of 2.7 billion people. This is a large proportion of the developing world’s population, especially when considering that these countries are major exporters of medicines to lower income countries (regardless of whether there any patents filings in importing countries). Therefore, just two patents could have an enormous impact. While this concerns an admittedly small subset of the MLEM, these patented medicines are of serious global public health interest in specific cases (e.g., curative treatments for hepatitis C) and should not be minimized [48]. Narrowly focused patent studies are useful for further evaluating these potential problem areas on a case-by-case basis [12–18].

We hope the way in which we have quantified our findings provide additional rationale for directing such targeted studies where more medicine patent filings are likely to be found consistently. These outcomes may also be useful for incorporating into economic development models or for future comparative studies. For example, how will a patent survey of the 2023 MLEM compare in terms of the number of developing countries typically covered by those patent estates? How would its findings compare in terms of the increases in population size, income, and health spending that are predictive of an increase of 1 additional essential medicine patent?

### Limitations

This study must be interpreted in light of its limitations. Indeed, others have critiqued our validation procedure with patent-holding companies [49, 50]. The objection is that these companies have a conflict of interest that may prevent them from reporting honestly. We feel, however, it would be a mistake to exclude companies willing to be transparent about their own international patent holdings. Further, it is unclear whether such a conflict of interest would lead companies to over- or under-report their patent holdings. Therefore, we have instead chosen to seek their participation and report upon the discrepancy between the datasets so readers can have a sense of what changes were made and the direction of these changes (i.e., whether the final dataset was smaller or larger). Another caveat about our study was that it did not distinguish between the patent types (e.g., patents on the chemical compound versus formulation patents) nor does it distinguish between the patent families. To compensate for this, we reported on the patent estates’ ages since newer patent estates likely include those upon the active ingredient while older ones are likely to pertain more to secondary aspects of the medicine, such as its formulation [51]. An additional limitation is that the presence of patents does not necessarily entail medicine access challenges; in fact, some studies have argued the opposite may be true—countries with lower levels of intellectual property protection may have fewer market launches and therefore lower access [8, 9]. Future studies linking procurement data with patent data would be able to investigate more directly the matter of whether there is an actual access problem attributable to a presence or absence of patents, rather than just the potential for one. Such studies would also ideally account for drug prices, for WTO membership, and for the use (or non-use) of TRIPS-flexibilities that allow for generic access even where patent-holding companies intend to enforce their patents rights (e.g., Least Developed Countries have until 2021 to bring their patent law to be TRIPS-compliant). Finally, this study focused on the 2013 MLEM because it was the most current list when the fieldwork was undertaken. As of publishing, two new editions of the MLEM will be available (the 2015 and 2017 editions). We have, therefore, focused this article on results that may be useful comparators, should future patent surveys like the current one be undertaken again. These MLEM updates will likely include larger samples of newer patented essential medicines [25], given advances in the treatment of HCV and in other areas.

## Conclusion

In this update to previous studies, we found that essential medicine patents were typically filed in countries with higher incomes, with higher healthcare expenditures, and especially with larger populations. Patent estates’ ages and the historical context from which they originated appeared to make a difference in the number of developing countries that they covered. We identified a time period (1995–2005) wherein there appeared to be elevated patent filing activity in the developing world. We hope future similar studies with other samples of medicines will be able to confirm or revise these findings (and build upon the methodology to address the limitations discussed above or to factor in medicine availability or prices). Broad surveillance and benchmarking of the global medicine patent landscape over time is valuable for detecting significant shifts that be occurring in patent filing trends and which may have implications for medicine access. With advances in international medicine patent transparency and patent data published by third parties, such studies will be increasingly feasible (especially considering the data made available through the Medicines Patent Pool [52]). To avoid previous confusion about calls for “patent transparency” [49, 50, 53], we are referring to asking companies to publicly disclose information on their international patent holdings (e.g., patent application numbers, expiration dates, the patents’ legal status); this is a separate issue from third party studies about companies’ patents using data obtained from other sources [35, 54]. When companies are proactively transparent about their patent holdings and about proactively facilitating equitable access in these cases, the stage is set for meaningful partnership and cooperation. We hope such transparency in the name of global public health will become the industry standard. Fortunately, some companies [55–57] are moving in this direction, including those who are now partnering with the Medicines Patent Pool [58] and score well on the Access to Medicines Index for transparency [59]. New partnerships like these may bring new avenues for facing the unprecedented challenges that will come with ensuring affordable access to the next generations of patented medicines that have been added to the MLEM.

## Additional files

Additional file 1. Methodological supplement. (DOCX 215 kb)

Additional file 2. Essential medicine patent data files. (XLSX 45 kb)

## Abbreviations

GDI: Gross domestic income
GDP: Gross domestic product
HDI: Human development index
HIV/AIDS: Human immunodeficiency virus infection and acquired immune deficiency syndrome
INPADOC: INternational PAtent DOCumentation
MLEM: Model list of essential medicines
TRIPS: Trade-related aspects of intellectual property rights
WHO: World Health Organization
WIPO: World intellectual property organization
WTO: World trade organization
