# Supplementary material for: In which developing countries are patents on essential medicines being filed?
Source: Global Health. 2017 Jun 26;13:38. doi: 10.1186/s12992-017-0262-4 (PMC5485610; doi:10.1186/s12992-017-0262-4)
Supplement: Supplementary file 1 — Methodological supplement. (DOCX 215 kb) [file 12992_2017_262_MOESM1_ESM.docx]

**Methodological Supplement**

*The following is reproduced with permission from the WIPO Report available (pages 7-8): Beall RF & A Attaran. 2016. Patent-based analysis of the World Health Organization’s 2013 Model List of Essential Medicines. Global Challenges Report, WIPO: Geneva. www.wipo.int/globalchallenges*

**Introduction**

The objective of this project is to determine, to the extent possible, which medicines on the 2013 WHO MLEM (18th edition) are patented, and where. A variety of approaches have been suggested and used for collecting international patent data globally on a single medicine, (UNDP 2012; Attaran 2004; Cavicchi and Kowalski 2009; Mackey 2012; WIPO 2011a; WIPO 2011b; Attaran and Gillespie-White 2001; Clark and Kowalski 2012) varying widely in cost and complexity. We opted to develop an approach that could be replicated in the future with each update of the MLEM, and one that builds upon the data and methodology of the studies that have preceded it, namely, Attaran’s report in 2004, and Cavicchi and Kowalski’s in 2009/11 (Attaran 2004; Cavicchi and Kowalski 2009, 2011). The fieldwork was undertaken in 2014/15 using the latest available edition of the MLEM (18th edition revised in 2013). This study was completed in three phases. See Figure 1. Firstly, we created a shortlist of MLEM medicines known or thought to be patented, separate from those which could be safely set aside as no longer covered by patent protection. Secondly, we determined the patent families for each medicine by using secondary sources of patent information from established databases. Thirdly, to verify or improve our tentative results, we contacted each medicine patent holder or supplier with our preliminary findings and requested their feedback and corrections, which all but two provided. This resulted in a validated patent portfolio for each medicine.

**PHASE 1: DETERMINING WHICH OF THE 375 MEDICINES ON THE 2013 MLEM ARE PATENTED**

Determining whether or not a given medicine is patented so as to preclude generic competition in a given country is a deceptively complicated question for a number of reasons. Firstly, there is no such thing as a “global patent”, meaning that patents in one country might be different or even non-existent in another country. Secondly, even where a patent exists, it may not be effective at blocking generic competition. For example, a patent on a medicine’s manufacturing process might be skirted by manufacturing it in some other way. Simply put, both the territory of the patents and the scope of the patent claims matter. Our approach addresses these ambiguities. Consistent with other studies (UNDP 2012; Milani and Oh 2011; Attaran 2004; Cavicchi and Kowalski 2009, 2011; Attaran and Gillespie-White 2001; Amin 2013), the United States and Canada were used as the base jurisdictions for the assessment of which medicines are patented. These countries represent much of the global pharmaceutical market (the United States alone represents 37 per cent of medicine sales globally in 2009) (UNDP 2012); they grant high numbers of patents annually; afford TRIPS-consistent patent protection; and require companies to transparently disclose patent holdings in the United States Food and Drug Administration’s Orange Book (FDA 2015b) and Purple Book (FDA 2015a), and Health Canada’s Patent Register (Health Canada 2015a). The Orange Book and Drug Product Database (Health Canada 2015b), respectively, also provide data on whether generic alternatives exist in the US and Canada. Checking for the presence of generic competition in the base jurisdictions is an advance on previous methodologies. As not all patents block generic competition, this extra step distinguishes those products for which there is a single source supplier from those having multiple generic suppliers already on the market despite patents being listed. Where these sources contain a patent disclosure for an MLEM medicine having the same drug, strength, formulation, and demonstrate an absence of generic competition for that medicine in the respective jurisdiction, that medicine was presumed to be patented elsewhere and included for further patent screening. We also consulted the data files from the previous patent studies on the MLEM that used similar methodologies (Attaran 2004; Cavicchi and Kowalski 2009, 2011) so as to exclude medicines whose patents had by now exceeded 21 years from the application filing date (full patent protection in most countries expires at 20 years). Applying these criteria, presumptively patented medicines for the purposes of this exercise are those that: (i) have patents listed in the United States or Canada; (ii) are available only in the originator form in the respective jurisdiction; and (iii) had not yet been determined to be post-patent by a previous study. The two medicines for which we were not able to apply this method, because they were not marketed in the US or Canada, were automatically included for the next phase.

**PHASE 2: DETERMINING WHERE MEDICINES ON THE 2013 MLEM ARE PATENTED IN DEVELOPING COUNTRIES**

With patent information for each of the medicines shortlisted as above, all related patent publications (including applications) – regardless of jurisdiction – were extracted from the INPADOC and Derwent databases using the Thomson Innovation portal (Thomson Reuters 2015). Each of these databases has international coverage and groups related patents and applications into families. INPADOC families are the most inclusive. They include all the documents directly or indirectly linked via a priority document (INPADOC 2008). Derwent families are more parsimonious because they are maintained by analysts who group entries according to a number of criteria (i.e., claims and applicants) stemming from a “basic application,” but still occasionally capture patents overlooked by INPADOC (Cavicchi and Kowalski 2009). These databases were used for the previous MLEM patent studies and for the purposes of the present one, data were combined and duplicates removed (Attaran 2004; Cavicchi and Kowalski 2009 and 2011). Each patent document (granted patents and applications) in our study is related to one of 88 Derwent or INPADOC extended families. Most pharmaceutical patents or applications describe one or more of the following innovations: a compound, a process, method of treatment, a formulation, or a co-formulation. Based on the title, abstract and patent claims, two reviewers independently classified each patent’s relevance under these categories. The Merck Index (Royal Society of Chemistry 2015) maintained by the Royal Society of Chemistry in Cambridge was further consulted for additional assistance in the classification exercise. We consider medicines with active compound patents to be of particular interest, since they are uniquely effective at establishing and maintaining market exclusivity for the patent holder, because they cover the medicine’s active ingredient. We tabulated the territorial coverage of each granted patent and patent application by country, except for countries categorized as “high-income” by the World Bank (World Bank 2015) or with “very high human development” by United Nations Development Program (UNDP 2014). These preliminary patent data were entered into a spreadsheet and prepared for verification by the suppliers during the next phase of the study.

**PHASE 3: PATENT DATA VERIFICATION**

To reduce the chances of error, each preliminary report was sent to the supplier company (or patent holder) for verification. This is an important step as it is ultimately the responsibility of the patent holder to apply for patents internationally, maintain those rights where granted, and enforce said rights when and where infringement occurs. The patent holders, therefore, are uniquely positioned to know where exactly they have rights on a particular product at a given time, and whether they intend to enforce those rights. It is also critical because the records of supplier companies may include records not located or not contained in the INPADOC and Derwent databases. This additional verification step is desirable and adds accuracy, but a fairly good picture of patent coverage can be obtained without it. A verification step was used in Attaran’s original study in 2004 (Attaran 2004), but not in the studies by Cavicchi and Kawolski in 2009 and 2011 (Cavicchi and Kowalski 2011). Companies were asked only for the minimum information necessary to confirm the existence of a patent: namely, patent or application numbers, expiration dates, and legal status in a given jurisdiction. No further information was required to complete the survey; however, space was provided for respondents, optionally, to note additional salient information such as voluntary patent non-enforcement, the presence of generic competition, voluntary licenses, access programs, etc. As most companies were initially reluctant or non-respondent, either the authors or WIPO made several attempts to contact them and elicit their cooperation. This verification process took seven months, after which all major companies decided to participate in the exercise, except for two (Cipla and InSite Vision).

**NOTES**

Access to Medicines Index 2014. Access to Medicines Index. Available from: [www.ow.ly/UXdgk](http://www.ow.ly/UXdgk)

Access to Medicines Index 2015. Access to Medicines Index: Ranking: Patents and Licensing. Available from: www.ow.ly/UXd6Q

Amin T. 2013. Patent Landscape Report For Pegylated Interferon Alfa 2A & 2B: Initiative for Medicines, Access & Knowledge (I-MAK). Available from: www.ow.ly/ UXcmG

Attaran A, L Gillespie-White 2001. Do patents for antiretroviral drugs constrain access to AIDS treatment in Africa? JAMA 286(15):1886-92.

Attaran A. 2004. How Do Patents And Economic Policies Affect Access To Essential Medicines In Developing Countries? Health Affairs, 23(3):155-66.

Beall RF, Kuhn R, Attaran A 2015. Compulsory Licensing Often Did Not Produce Lower Prices For Antiretrovirals Compared To International Procurement. Health Affairs.;34(3):493-501.

Bigdeli M, B. Jacobs, G. Tomson, R. Laing, A. Ghaffar, B. Dujardin, et al. 2013. Access to medicines from a health system perspective. Health policy and planning. 28(7):692-704.

Brinckerhoff CC, Schorr K. 2015. Patent watch: Have the biosimilar floodgates been opened in the United States? Nat Rev Drug Discov.;14(5):303-4.

Cavicchi JR, SP Kowalski 2009. Report of Patent Literature, Search Methodology and Patent Status of Medicines on the WHO EML. International Technology Transfer Institute, Franklin Pierce Center for Intellectual Property.

Cavicchi JR, SP Kowalski 2011. Preliminary Report on Search Methodology and Patent Status of Medicines Added to the WHO EML from the 18th meeting of the WHO Expert Committee on the Selection and Use of Essential Medicines. University of New Hampshire.

Clark KL, SP Kowalski 2012. Harnessing the power of patent information to accelerate innovation. Wiley Interdisciplinary Reviews: Data Mining and Knowledge Discovery 2(5):427-35.

European Patent Office 2008. The “extended” (INPADOC) patent family. Available from: www.ow.ly/UXcAW

Friedman MA, den Besten H, Attaran A. 2003. Out-licensing: a practical approach for improvement of access to medicines in poor countries. The Lancet.;361(9354):341-4.

Global Fund 2015. Price and Quality Reporting. Available from: www.ow.ly/UXbTq

Health Canada 2015. Drug Product Database Online Query. Available from: www.ow.ly/UXcvv

Health Canada 2015. Patent Register. Available from: www.ow.ly/UXbFK88

Laing R, B Waning, A Gray, N Ford, E t’Hoen, 2003. 25 years of the WHO essential medicines lists: progress and challenges. The Lancet.361(9370):1723-9.

Landon IP. Patent Landscape Report on Ritonavir Geneva, Switzerland: World Intellectual Property Organization 2011. Available from: www.ow.ly/UXci919

Mackey TK, BA Liang 2012. Patent and Exclusivity Status of Essential Medicines for Non-Communicable Disease. PLoS ONE. 7(11):e51022.

Medicines Patent Pool 2015. Licences in the MPP. Available from: www.ow.ly/UXcYJ Merck 2015.

Merck US patent rights for products. Available from: www.ow.ly/UXcVy

Milani B., C. Oh 2011. Searching for patents on essential medicines in developing countries: a methodology. International Journal of Intellectual Property Management. 4(3):191-209.

Royal Society of Chemistry 2015. The Merck Index. Available from: www.ow.ly/UXcHM

t’Hoen E 2014. Patent Status Information and Public Health. World Health Organization, (t’Hoen 2014).

Thomson Reuters 2011 IP Solutions ICG. Patent Landscape Report on Atazanavir: World Intellectual Property Organization. Available from: www.ow.ly/UXcbc

Thomson Reuters 2015. Thomson Innovation. Available from: www.ow.ly/UXcyz

United Nations Development Programme 2012. Patent Information and Transparency: A Methodology for Patent Searches on Essential Medicines in Developing Countries New York, NY: United Nations Development Programme. Available from: www.ow.ly UT3P8 p—28

United Nations Development Programme 2014. Data - Human Development Reports. Available from: www.ow.ly/UXdzZ

United States Food and Drug Administration 2015. Orange Book: Approved Drug Products with Therapeutic Equivalence Evaluations. Available from: www.ow.ly/UXcrr

United States Food and Drug Administration 2015. Purple Book: Lists of Licensed Biological Products with Reference Product Exclusivity and Biosimilarity or Interchangeability Evaluations. Available from: www.ow.ly/UXdtz

World Bank 2015. Data - Country and Lending Groups. Available from: www.ow.ly/UXcM2 (World Bank 2015)

World Health Organization 2004. Equitable access to essential medicines: a framework for collective action. Available from: www.ow.ly/UXdbb

World Health Organization 2013. WHO Model List of Essential Medicines - 18th list. Available from: www.ow.ly/UXdTX World Health Organization 2015.

WHO Model List of Essential Medicines - 19th List. Available from: www.ow.ly/UXcSY

World Health Organization 2015. Essential Medicines Selection. Available from: www.ow.ly/UXbZX

World Health Organization 2015. HIV/AIDS: Global Price Reporting Mechanism for HIV, tuberculosis and malaria. Available from: www.ow.ly/UXc5t

World Trade Organization 2001. Declaration on the TRIPS Agreement and public health. Available from: www.ow.ly/UT3x8

World Trade Organization 2006. Trips and Health: Frequently Asked Questions: Compulsory licensing of pharmaceuticals and TRIPS. Available from: www.ow.ly/UXdkb

World Trade Organization 2015. WHO, WIPO, WTO Trilateral Cooperation on Public Health, IP and Trade. Available from: www.ow.ly/UXd2R

Yamane H. 2011. Interpreting TRIPS: globalisation of intellectual property rights and access to medicines: Bloomsbury Publishing

**Exclusions**

- Rich countries

- Patents older than 21 yrs

**Figure 1. Workflow for compiling MLEM product selection and patent compilation**

Health Canada

-Patents?

-Generics?

INPADOC extended patent families

Derwent World Patent Index families

Merged via Thomson Innovation

Preliminary reports for each included medicine

USFDA Orange Book

-Patents?

-Generics?

Previous studies

-

Additions to 2013 MLEM

*1. Medicine/*

*product selection*

*2. Domestic to int’l patent family linkage*

Patent estate reports sent to suppliers

18

validated reports

2

un-validated reports

*3. Validation by originator companies*

Final dataset

Concordance study

Duplicates removed

Outdated data removed
